# Anp32e promotes renal interstitial fibrosis by upregulating the expression of fibrosis-related proteins

**DOI:** 10.7150/ijbs.74431

**Published:** 2022-09-25

**Authors:** Ju Wei, Yi Shan, Zheng Xiao, Lu Wen, Yilin Tao, Xi Fang, Hanwen Luo, Chengyuan Tang, Ying Li

**Affiliations:** 1 Department of Nephrology, The Second Xiangya Hospital of Central South University, Changsha, 410011, Hunan, China.; 2 Hunan Key Laboratory of Kidney Disease and Blood Purification, Changsha, 410011, Hunan, China.

**Keywords:** Anp32e, renal interstitial fibrosis, CKD, fibrosis-related protein, pro-fibrotic factor, TGF-β1/Smad3

## Abstract

Acidic nuclear phosphoprotein 32 family member e (Anp32e) has been reported to contribute to early mammalian development and cancer metastasis. However, the pathophysiological role of Anp32e in renal interstitial fibrosis (RIF) is poorly understood. Here, we demonstrated that Anp32e was highly expressed in the region of RIF in patients with IgA nephropathy, unilateral ureteral obstruction (UUO) mouse kidneys, and Boston University mouse proximal tubular (BUMPT) cells when treated with TGF-β1; this upregulation was positively correlated with the total fibrotic area of the kidneys. The overexpression of Anp32e enhanced the TGF-β1-induced production of fibrosis-related proteins (fibronectin (Fn) and collagen type I (Col-I)) in BUMPT cells whereas the knockdown of Anp32e suppressed the deposition of these fibrosis-related proteins in UUO mice and TGF-β1-stimulated BUMPT cells. In particular, Anp32e overexpression alone induced the deposition of Fn and Col-I in both mouse kidneys and BUMPT cells without TGF-β1 stimulation. Furthermore, we revealed that the overexpression of Anp32e induced the expression of TGF-β1 and p-Smad3 while TGF-β1 inhibitor SB431542 reversed the Anp32e-induced upregulation of Fn and Col-I in BUMPT cells without TGF-β1 stimulation. Collectively, our data demonstrate that Anp32e promotes the deposition of fibrosis-related proteins by regulating the TGF-β1/Smad3 pathway.

## Introduction

Renal interstitial fibrosis (RIF) is the hallmark of progressive renal diseases of virtually any etiology and can progress to chronic kidney disease (CKD) and then to end-stage renal disease (ESRD) 1, 2. The severity of RIF has been shown to be positively correlated with the decline in renal function and can be used to evaluate the progression and prognosis of CKD 3, 4. Therefore, the suppression of RIF is a promising strategy for preventing CKD development. RIF is characterized by the deposition of fibrosis-related proteins in the renal cortex, including fibronectin (Fn) and collagen type I (Col-I) 1. Although canonical signaling pathways, such as the TGF-β/Smad, Wnt/β-catenin and c-Jun amino terminal kinase pathways are known to play important roles in RIF 1, 3, 5, the specific molecular mechanisms underlying RIF still remain unclear.

Acidic nuclear phosphoprotein 32 family member e (Anp32e) (also known as Lanp-L and Cpd-1) is a member of the Anp32 protein family with multiple biological functions, including chromatin modification and remodeling, gene regulation, protein phosphorylation and the regulation of intracellular transport 6-8. It has been reported that Anp32e is not only vital for cerebellar development and synaptogenesis but also participates in the development of tumors 9-12. In addition, Anp32e was found to be highly expressed in both pancreatic and thyroid cancer cells and promoted the proliferation and migration of tumor cells 13, 14. Interestingly, in breast cancer cells, knockdown of Anp32e inhibited EMT, which plays a pivotal role in progressive renal interstitial fibrosis^15^, ^16^. Thus, the role of Anp32e in RIF deserves further investigation.

In this study, we detected the expression of Anp32e protein in patients with IgA nephropathy (IgAN), unilateral ureteral obstruction (UUO) mice, and Boston University mouse proximal tubular (BUMPT) cells stimulated by TGF-β1. We also investigated the role of Anp32e protein in the enhanced deposition of renal fibrosis-related proteins (Fn, Col-I) both *in vivo* and *in vitro*. Finally, we found that the overexpression of Anp32e alone could induce the deposition of fibrosis-related proteins by activating the TGF-β1/Smad3 signaling pathway.

## Materials and methods

### Selection Patients

We recruited patients with IgAN who had been hospitalized in the Department of Nephrology at the Second Xiangya Hospital from 2020 to 2021. The inclusion criteria were as follows: (1) age ≥18 years; (2) patients who underwent renal biopsy and were confirmed to have RIF as demonstrated by Masson's trichrome staining; and (3) good compliance with clinical instructions. The exclusion criteria were as follows: (1) patients with contraindications for renal biopsy; (2) patients who were complicated by other serious diseases including serious cardiovascular disease, chronic hepatitis B active phase, and malignant tumor; (3) other secondary kidney diseases, such as diabetes, hypertension, systemic lupus, viral hepatitis B, Henoch-Schonlein purpura, multiple myeloma, thyroid and other tumors, were excluded by renal biopsy or clinical treatment; (3) those who showed poor compliance with clinical tests; and (4) patients who did not provide informed consent.

Patients underwent renal carcinoma resection in the Department of Urology at Second Xiangya Hospital were selected as the control group. The inclusion criteria were as follows: (1) age ≥18 years of either gender; (2) nephrectomy was performed and staining of the adjacent renal tissue with Masson's trichrome did not demonstrate RIF; (3) no other renal diseases were found. The exclusion criteria were as follows: (1) complicated with other serious diseases; (2) poor compliance to clinical tests; and (3) failure to provide informed consent.

### Animal experiments

Male C57BL/6 mice (8-12 weeks-of-age), weighing from 20-25g, were purchased from Hunan Slack King Experimental Animal Company (Changsha, China). The mice were randomly divided into several groups and housed in the animal facility at the Second Xiangya Hospital with free access to water and food.

### Construction of the UUO mouse model

First, mice were anesthetized with pentobarbital (60 mg/kg). Then, the left kidney and ureter were exposed through an abdominal midline incision. The left ureter was separated and ligated at the middle and upper one-third of the ureter near the hilum and ligated again far from the hilum. The ureter was clipped in the middle of the two ligation points, and the ureter and the kidney were backed to the abdomen. The peritoneum, muscle, and subcutaneous tissue were sutured successively, and the skin was clipped. Then, 1 mL of sterile saline was subcutaneously injected into the back of each mouse for fluid resuscitation. The mice were then placed on a heated mat to rewarm and were returned to their normal environment once they had woken up and regained vitality.

### Lentiviral vector construction

Anp32e overexpression and knockdown lentiviruses were constructed by GeneChem Company (Shanghai, China). For the overexpression lentivirus (LV-Anp32e), we obtained the full length Anp32e (NM_023210) from NCBI and inserted this into a vector (Ubi-MCS-3FLAG-CBh-gcGFP-IRES-puromycin) to form a recombinant plasmid; this was packaged with the virus and purified. For the knockdown lentivirus (sh-Anp32e), we designed and synthesized Anp32e shRNA (GCTATGAAGAGGAGGAAGA). The shRNA was combined with a vector (hU6-MCS-CBh-gcGFP-IRES-puromycin), packaged with the virus and then purified.

### Intrarenal injections

The lentiviral vectors were delivered into the kidneys of experimental mice by intrarenal injection 17, 18. To establish Anp32e overexpressed mice, 100 µL of purified LV-Anp32e (2 × 10^8^ TU/ml), LV-NC or saline was injected into the left kidney. To establish the Anp32e knockdown mice, 85 µL of purified sh-Anp32e (3.5 × 10^8^ TU/ml), scramble-shRNA (Scr shRNA) or saline was injected into left kidney.

Mice were anesthetized with pentobarbital and then fixed to expose the left kidney. Purified lentivirus was extracted with a 1 ml syringe connected to a 25-G needle. The control group was injected with the same amount of sterile normal saline. The needle was inserted from the lower pole of the kidney along the longitudinal axis and carefully pushed to the upper pole at a depth of approximately 0.5 cm, avoiding renal veins, arteries and the ureters. Then, the needle was slowly withdrawn and injected at the same time. The kidney turned white after the injection; which indicated that the injection was successful. We then waited 2-3 seconds before removing the needle. Finally, the temperature of the mice was restored with the help of an electric blanket and a heater. The kidneys of the mice receiving the Anp32e overexpression lentivirus were harvested 2 weeks after injection. For the Anp32e knockdown lentivirus mice, UUO surgery was performed 1 week after injection and the mice were sacrificed 1 week thereafter.

### Cell culture and treatment

The Boston University mouse proximal tubular (BUMPT) cell line was provided by Dr. Z. Dong at the Second Xiangya Hospital of Central South University 19. The cells were cultured in Dulbecco's Modified Eagle's Medium (DMEM) (Gibco; Thermo Fisher Scientific, Waltham, MA, United States) with 10% fetal bovine serum (Biological Industries, Cromwell, CT, United States), 1% sodium pyruvate (Gibco), 1% penicillin-streptomycin (Gibco) and 1% L-Glutamine (Gibco). To establish a TGF-β1-induced fibrosis model, cells were pre-incubated in serum-free culture medium at 37°C in a humidified atmosphere of 5% CO_2_ for 12-18 hours and stimulated with different TGF-β1 (PeproTech, Rocky, Hill, NJ, United States) concentrations (0, 2.5, 5, 10ng/ml) for 24hours or 5 ng/ml TGF-β1 for different stimulation times (0, 0.5, 1, 2, 6, 12, 24, 30 hours). Subsequently, to inhibit the TGF-β1/Smad3 signaling pathway, BUMPT cells were treated with 10µM type I TGF-β1 receptor inhibitor SB431542 for 24 hours after transfection with Anp32e overexpression plasmids for 12 hours. (APExBIO, Houston, TX, United States).

### Small interfering RNA, plasmids and cell transfection

To overexpress or silence Anp32e, BUMPT cells at 60-80% confluency was transfected with the Anp32e-overexpression plasmid (Exp-Anp32e, GeneChem Company) or the Anp32e small interfering RNA (siRNA, RiboBio, Guangzhou, China), respectively. Transformation was carried out using lipofectamine 2000 (Invitrogen, Carlsbad, CA, United States) in accordance with the manufacturer's instructions. The medium was changed 6 hours later, and the cells were maintained for a further 24-48 hours. To judge the transfection efficiency of the plasmid, cells were observed with a fluorescence microscopy (Leica DMI 3000 B, Leica, Wetzlar, Germany), and 6-10 fields were randomly selected to determine the area of green fluorescence; using this data, we determined the mean transfection efficiency.

### Hematoxylin-eosin (HE) and Masson's trichrome staining

#### HE staining and tubular atrophy score

Kidney tissues were fixed with 4% paraformaldehyde, embedded in paraffin and sectioned at 4 µm. Then, the sections were stained with hematoxylin and ethanol/eosin reagents, fixed with neutral gum, and then analyzed by light microscopy (Leica DMI 3000 B, Leica, Wetzlar, Germany). Renal tubules with the following histopathological changes were considered to exhibit tubular atrophy: tubular epithelial thinning, pyknotic nuclei or tubular dilation, expansion of the interstitial space, with or without protein casts. Tubular atrophy was examined in a blinded manner and scored by the proportion (%) of atrophic tubules: 0, no damage; 1, < 25%; 2, 25-50%; 3, 50-75%; 4, > 75%. For quantification, at least 10 randomly selected fields per mouse and six mice for each group were scored.

#### Masson's trichrome staining

All samples were first stained with hematoxylin for 5 minutes, treated with acid ponceau magenta solution for 8 minutes, stained with aniline blue for 5 minutes, and then sealed with neutral gum. Finally, six fields were randomly selected under light microscopy. Then, we measured the area on each section that was occupied by collagen fibers.

### Immunohistochemistry (IHC) staining

Paraffin-embedded kidney sections (3-5 µm thick) were deparaffinized and incubated with 0.1 M sodium citrate, pH 6.0 at 65°C for 1 hour to perform antigen retrieval. Subsequently, IHC assays were performed with a SP kit (CW2069S, CWBIO, Jiangsu, China). All sections were exposed to an optimal dilution of solution containing anti-Anp32e antibody (ab5993, 1/50, Abcam, Cambridge, United Kingdom), anti-Fn antibody (ab2413, 1/200, Abcam), anti-Col-I antibody (AF7001, 1/200, Affinity Biosciences, United States), anti-TGF-β1 antibody (ab215715, 1/100, Abcam) and anti-p-Smad3 antibody (ab52903, 1/50, Abcam) respectively. Antibody diluent was used as a negative control to replace the primary antibodies. Image-Pro Plus 6.0 (Media Cybernetics, Bethesda, MD, United States) software was used to semi-quantitatively analyze all images; results are represented as the area of positive antibody binding.

### Western blotting

Frozen kidney tissues were lysed by Radio-Immunoprecipitation Assay (RIPA) buffer (Beyotime, Shanghai, China) and Protease Inhibitor Cocktail (CWBIO). Next, tissue and cell lysates were centrifuged separately at 13,523 g and 4°C for 15 minutes to obtain total protein extracts in the supernatant. A Bicinchoninic Acid (BCA) Protein Quantitation Kit (Beyotime) was used to detect protein concentration. Then, equal amounts (30 µg) of proteins from each sample were separated by 10% sodium dodecyl sulfate-polyacrylamide gel electrophoresis (SDS-PAGE) and transferred onto polyvinylidene difluoride membranes (Millipore, MA, United States). The membranes were blocked in 5% bovine serum albumin (BSA) (Beyotime) for 1 hour and then incubated overnight at 4°C with primary antibodies. The next day, membranes were incubated with horseradish peroxidase‐conjugated goat antirabbit immunoglobulin G (SA00001-2, 1/4000; Proteintech, Wuhan, China), a secondary antibody, for 1 hour. Finally, the proteins bound on the membranes were detected by ECL western blotting chemiluminescence reagents (Millipore). The following primary antibodies were used in this experiment: anti-Anp32e antibodies (ab5993, 1/1000; Abcam), Fn antibodies (ab2413, 1/1500; Abcam), Col-I antibodies (AF7001, 1/1000; Affinity Biosciences), TGF-β1(ab215715, 1/1000; Abcam), p-Smad3 (ab52903, 1/1000; Abcam), t-Smad3 (9523S, 1/1000; Cell Signaling Technology, Danvers, MA, United States), GAPDH (10494-1-AP, 1/3000; Proteintech), β-actin (20536-1-AP, 1/3000; Proteintech). Band intensity was evaluated by Image-Pro Plus 6.0 software.

### Quantitative real-time PCR (qRT-PCR)

Trizol reagent (Invitrogen) was used to extract total RNAs. The A260/A280 ratio was used to evaluate the purity of the RNAs. Synthesis of first strand cDNA was carried out using 2 µg of RNA in a 20 µl reverse transcription reaction and with a Prime Script kit (Takara, Tokyo, Japan). Reverse transcription was carried out in an automated thermal cycler at 37 ℃ for 15 minutes and then terminated at 85 ℃ for 30 seconds. Quantitative real-time PCR was performed with a LightCycler 480 Systems using SYBR Premix EX Tad (Takara). The PCR parameters were as follows: 95 °C for 30 seconds, followed by 40 cycles of amplification at 95 °C for 5 seconds and 60 °C for 30 seconds, with cooling at 50 ℃ for 30 seconds at the end. Relative mRNA levels were determined by fluorescent quantitative PCR and normalized by β-actin levels. Quantitative PCR results were then calculated using the 2^-ΔΔCt^ method 20. Specific primers for the use of SYBR Green are given in Table 1.

### Statistical analysis

The quantitative data shown in this study are representative of at least three experiments and are expressed as means ± standard deviation (SD). Statistical analysis was performed using GraphPad Prism software. One-way analysis of variance (ANOVA), followed by Tukey's post-tests, was conducted to identify statistically significant differences among groups. P<0.05 was significant.

## Results

### Anp32e was upregulated in IgAN patients with RIF

Renal biopsy tissues were collected from nine patients with IgAN. We also collected para-cancer tissues from seven patients underwent nephrectomy as controls. There were no significant differences in age or gender between the two groups. Statistical analysis showed that blood urea nitrogen, creatinine and the case of proteinuria in the IgAN group were significantly higher than those in the control group (Table 2). However, there was no significant difference in the level of blood uric acid when compared between the two groups.

**Figure 1:**
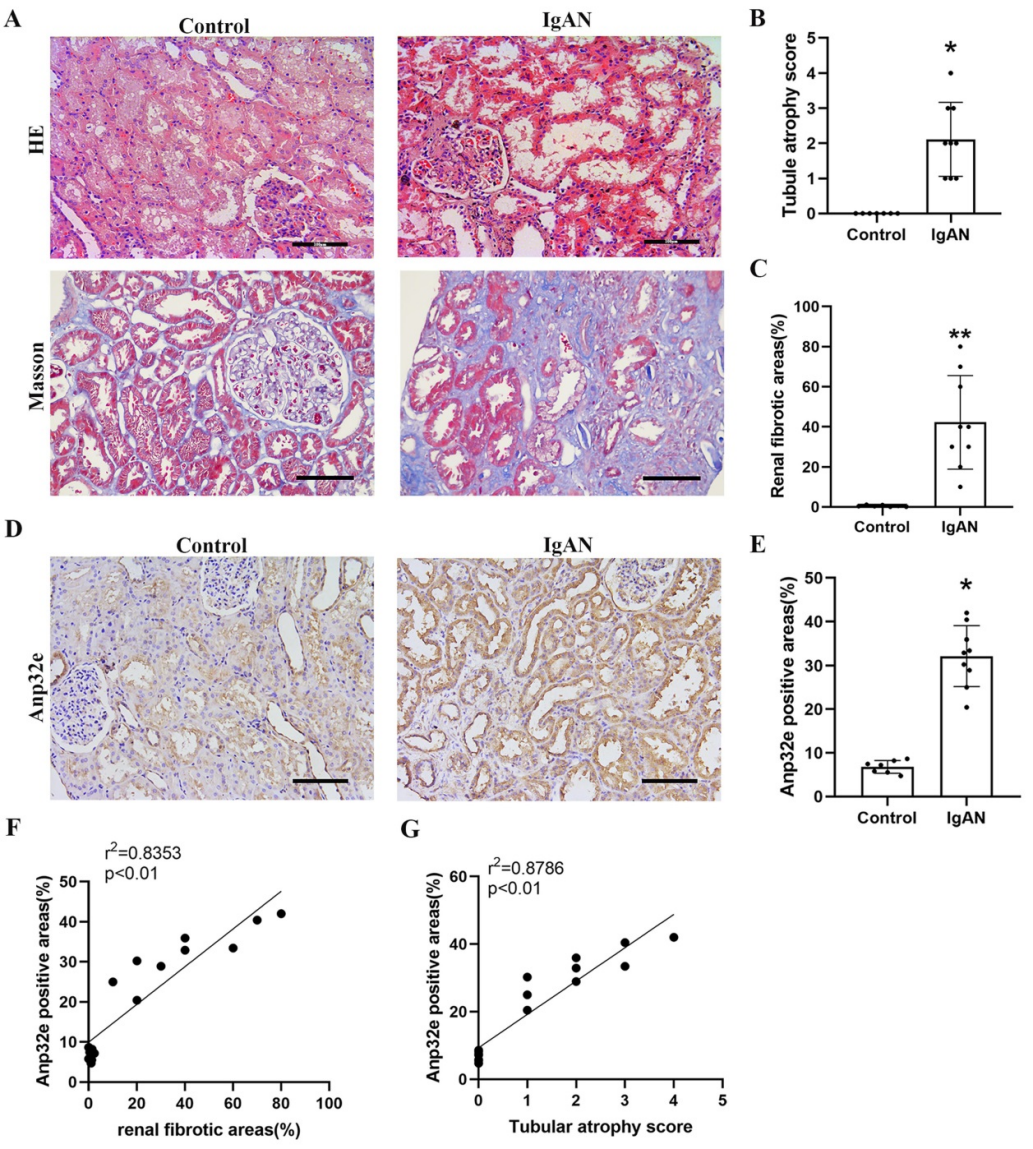
** The expression levels of Anp32e protein were upregulated in IgAN patients with RIF. (A)** HE and Masson trichrome staining in renal tissue from the two groups of patients (×200). Scale bar, 100 μm. **(B)** Tubular atrophy score (n=7/9). Tubular atrophy was graded as 0, 1 (1%-5%), 2 (26%-50%), 3 (51%-75%), and 4 (76%-100%) with regards to the proportion of tubules showing atrophy. **(C)** Renal fibrotic areas (%) in two groups (n=7/9). **(D)** IHC analysis of Anp32e in renal tissue from the two groups of patients (×200). Scale bar, 100 μm. **(E)** The positive areas (%) of Anp32e protein in renal tissue from the two groups of patients (n=7/9). **(F)** Linear regression between Anp32e and renal fibrotic areas (n=7/9). **(G)** Linear regression between Anp32e and renal tubular atrophy score (n=7/9). The correlation coefficient (r^2^) is shown in the upper left corner of the graph.^ *^P < 0.05, ^**^P < 0.01 *vs.* Control group. Data represent means ± SD**.**

Compared with the control group, HE and Masson's trichrome staining revealed significant tubule epithelial cell atrophy, tubule dilatation, protein casts, and collagen deposition in the IgAN group (Figure 1A-C). IHC analysis indicated that Anp32e protein was expressed in the renal tubular epithelial cells of IgAN patients and presented a much higher level than that in the control group (Figure 1D, E). Linear regression analysis of IHC and Masson's trichrome staining revealed a positive correlation between the areas of Anp32e expression and renal fibrosis (Figure 1F).

### Anp32e was upregulated in UUO mice and positively correlated with fibrosis-related proteins

**Figure 2:**
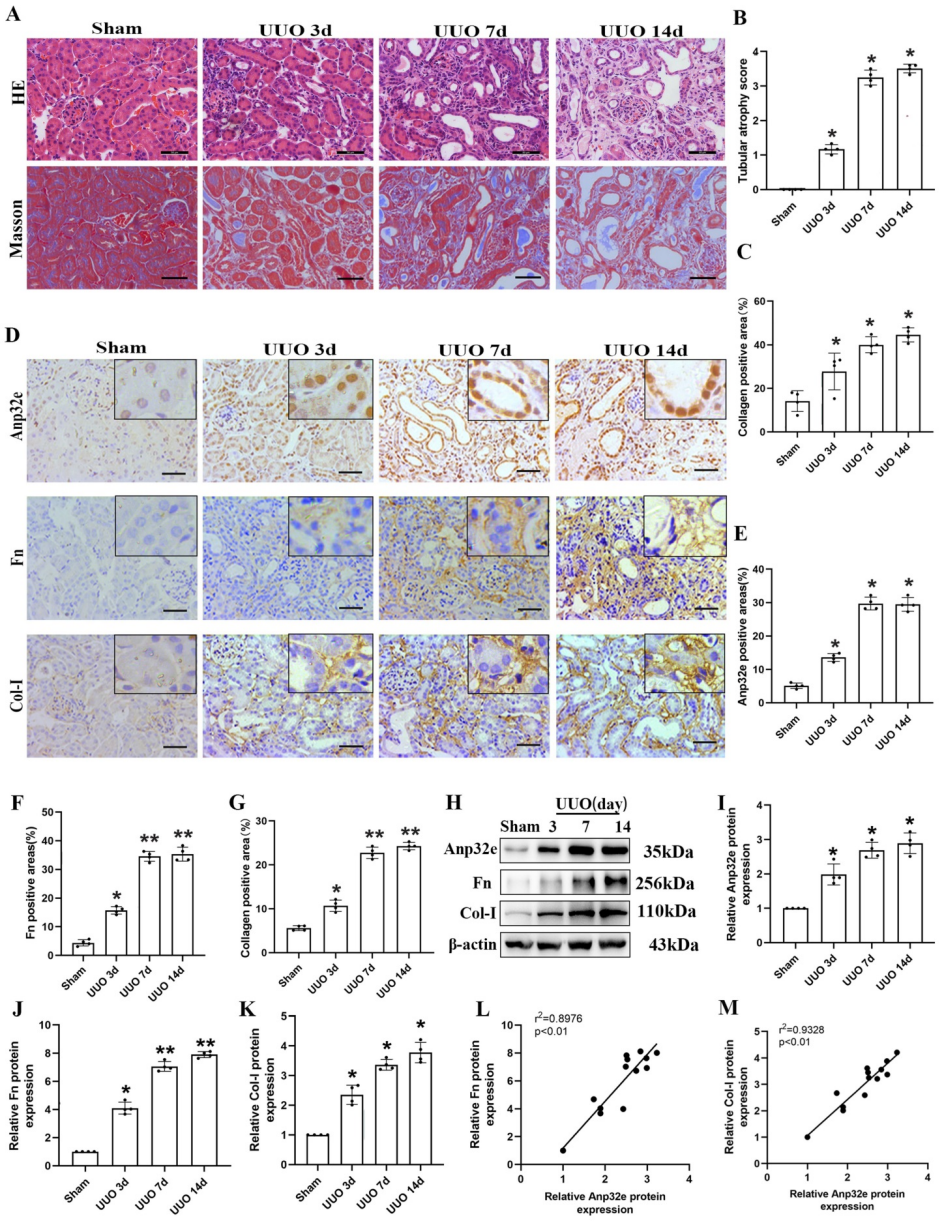
** The expression of Anp32e protein was upregulated and positively correlated with fibrosis-related proteins in UUO mice. (A)** HE and Masson trichrome staining in mice kidneys at different timepoints (×400). Scale bar, 50 μm. **(B)** Tubular atrophy score (n=4). **(C)** The positive areas (%) of collagen in various groups (n=4).** (D-G)** IHC analysis and the positive areas (%) of Anp32e, Fn, and Col-I in mice kidneys at different timepoints (×400). Scale bar, 50 μm, (n=4). **(H-K)** Western blotting and densitometry analysis of Anp32e, Fn, and Col-I proteins in various groups (n=4).** (L, M)** Linear regression analysis of western blotting results (n=4). The correlation coefficient (r^2^) is shown in the upper left corner of the graph.^ *^P < 0.05,^ **^P < 0.01 *vs*. sham group. Data represent means ±SD.

To clarify the role of Anp32e in RIF in the animal model, we harvested kidney tissues for pathological and biochemical analysis at 3, 7 and 14 days after UUO operation. HE and Masson's trichrome staining showed that kidneys from UUO mice exhibited loss of brush border, tubular dilation, tubular atrophy, and collagen deposition, in a time-dependent manner when compared with the sham group (Figure 2A-C). Moreover, IHC staining demonstrated that Anp32e protein was expressed in the glomeruli, renal tubules, and the renal interstitium in the sham group; however, the expression of Anp32e protein was significantly higher in the renal tubules of UUO mice in a time-dependent manner (Figure 2D-G). In addition, in line with our IHC results, the expression of Anp32e, Fn and Col-I proteins were consistently upregulated in a time-dependent manner, as determined by western blotting (Figure 2H-K). Furthermore, linear regression of western blotting analysis revealed a positive correlation between the expression levels of Anp32e and those of Fn and Col-I (Figure 2L, M).

### Anp32e was upregulated in TGF-β1 stimulated BUMPT cells

**Figure 3:**
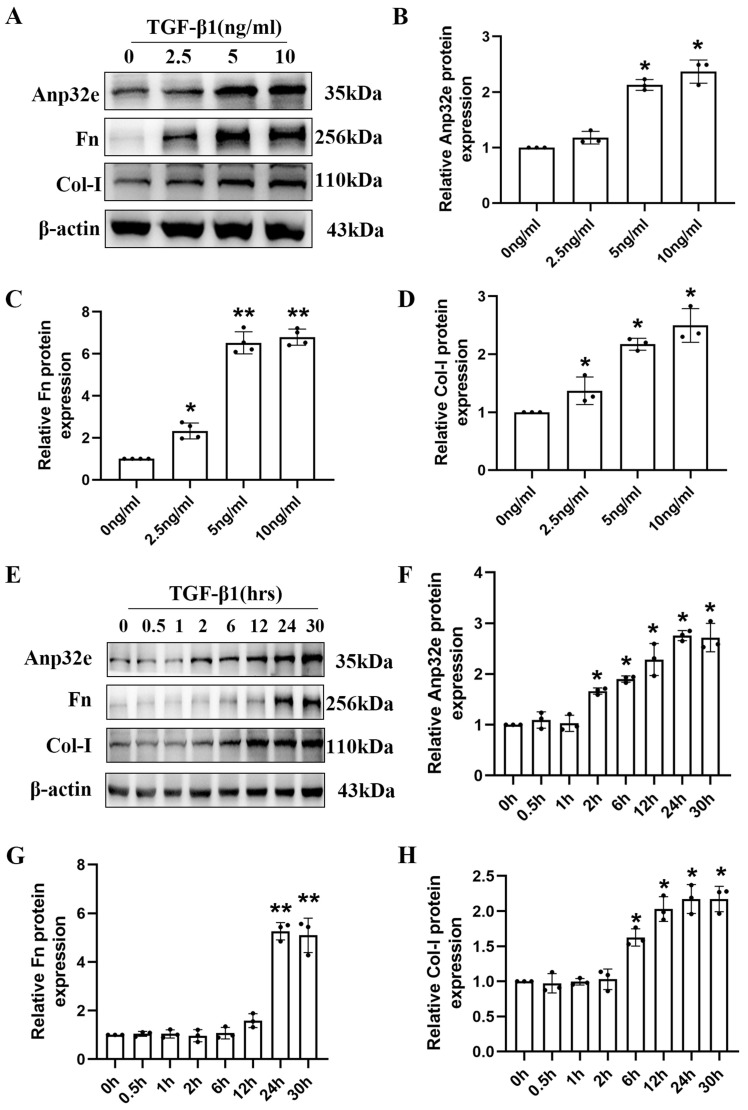
** The expression of Anp32e and fibrosis-related proteins was induced in TGF-β1 stimulated BUMPT cells. (A-D)** Western blotting and densitometry analysis of Anp32e, Fn, and Col-I in BUMPT cells treated with different concentrations of TGF-β1 (n=3).** (E-H)** Western blotting and densitometry analysis of Anp32e, Fn, and Col-I in BUMPT cells treated at different timepoints with TGF-β1 (n=3). ^*^P<0.05, ^**^P<0.01 *vs.* control group (0 ng/ml TGF-β1 group and 0 hours TGF-β1 group). Data represent means ± SD.

To further clarify the role of Anp32e protein *in vitro*, we constructed a kidney fibrosis model by treating BUMPT cells with TGF-β1. At first, BUMPT cells were treated with increasing concentrations of TGF-β1 (2.5, 5, and 10 ng/ml) for 24h; cells in the control group were not treated with TGF-β1. Western blotting revealed a dose-dependent increase in the expression of Anp32e, Fn and Col-I proteins (Figure 3A-D). Subsequently, BUMPT cells were treated with 5 ng/ml TGF-β1 for different time periods (0, 0.5, 1, 2, 6, 12, 24, and 30 hours). Western blotting showed that the expression of Anp32e, Fn, and Col-I proteins increased in a time-dependent manner (Figure 3E-H). Collectively, these results indicated that Anp32e may play an important promoting role in RIF.

### The overexpression of Anp32e enhanced the expression of fibrosis-related proteins in TGF-β1 stimulated BUMPT cells

**Figure 4:**
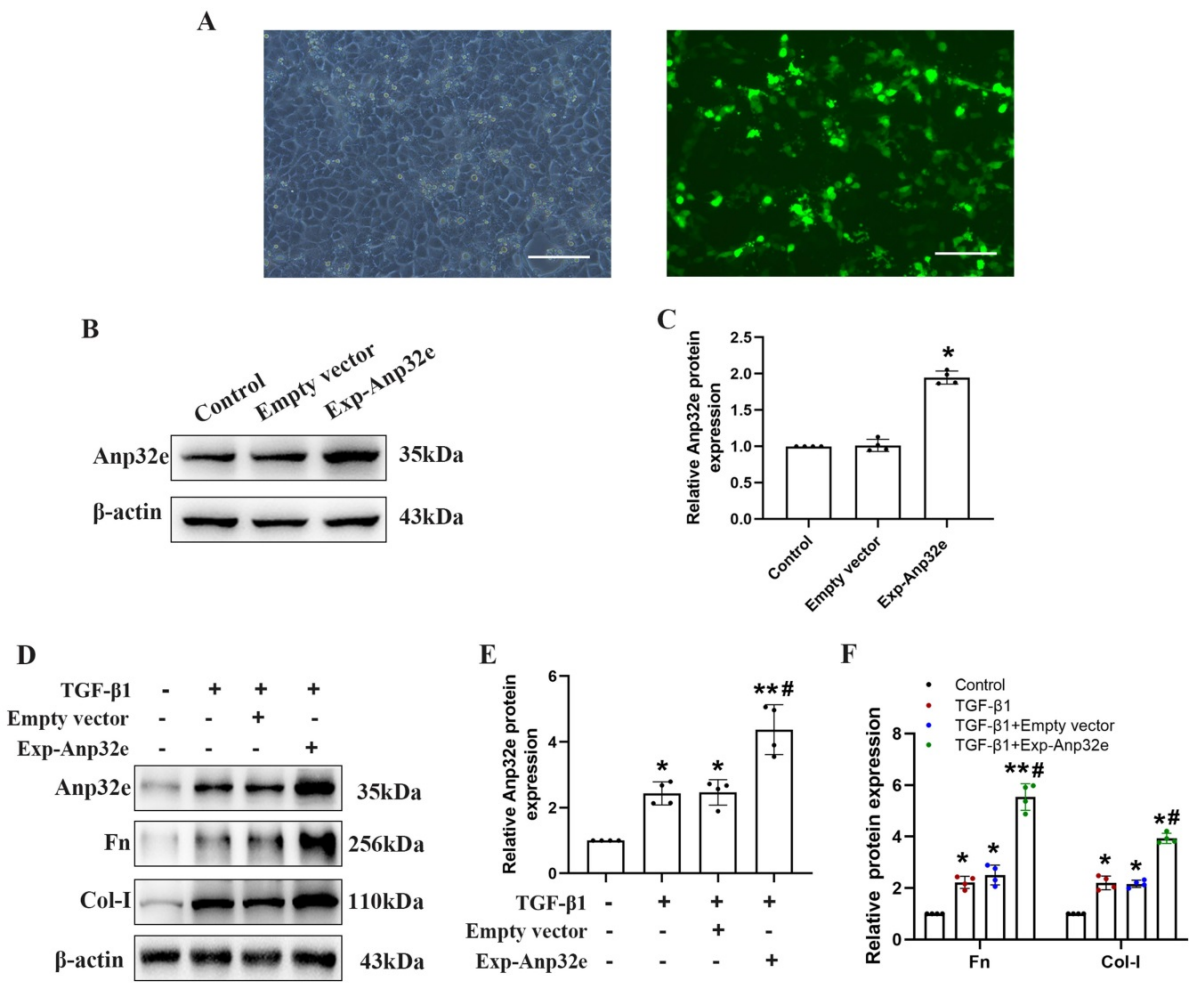
** Anp32e enhanced the expression of fibrosis-related proteins in TGF-β1 stimulated BUMPT cells. (A)** Representative fluorescence microscopy pictures showing the number of BUMPT cells exhibiting intracellular green fluorescence after transfection with the Exp-Anp32e plasmid (×200). Scale bar, 100μm. **(B, C)** Western blotting and densitometry analysis of Anp32e protein in BUMPT cells transfected with Empty vector or the Exp-Anp32e plasmid (n=4). **(D-F)** Western blotting and densitometry analysis of Anp32e, Fn and Col-I proteins in BUMPT cells transfected with Empty vector or Exp-Anp32e, and then treated with or without TGF-β1 (n=4).^ *^P < 0.05,^ **^P < 0.01 *vs.* control group and ^#^P < 0.05 *vs*. Empty vector + TGF-β1 group. Data represent means ± SD.

To investigate whether Anp32e regulates RIF, we overexpressed Anp32e in BUMPT cells. Fluorescence microscopy showed that more than 60% of the Exp-Anp32e-transfected cells exhibited green fluorescence (Figure 4A). Western blotting showed that Anp32e was overexpressed in BUMPT cells in a very efficient manner (Figure 4B, C). Furthermore, we also used 5 ng/ml of TGF-β1 to stimulate BUMPT cells 24 h after transfection; we found that Anp32e enhanced the expression of Fn and Col-I proteins in TGF-β1 induced BUMPT cells, as shown by western blotting (Figure 4D-F).

### Anp32e knockdown reduced the deposition of fibrosis-related proteins in UUO mice and TGF-β1 stimulated BUMPT cells

**Figure 5:**
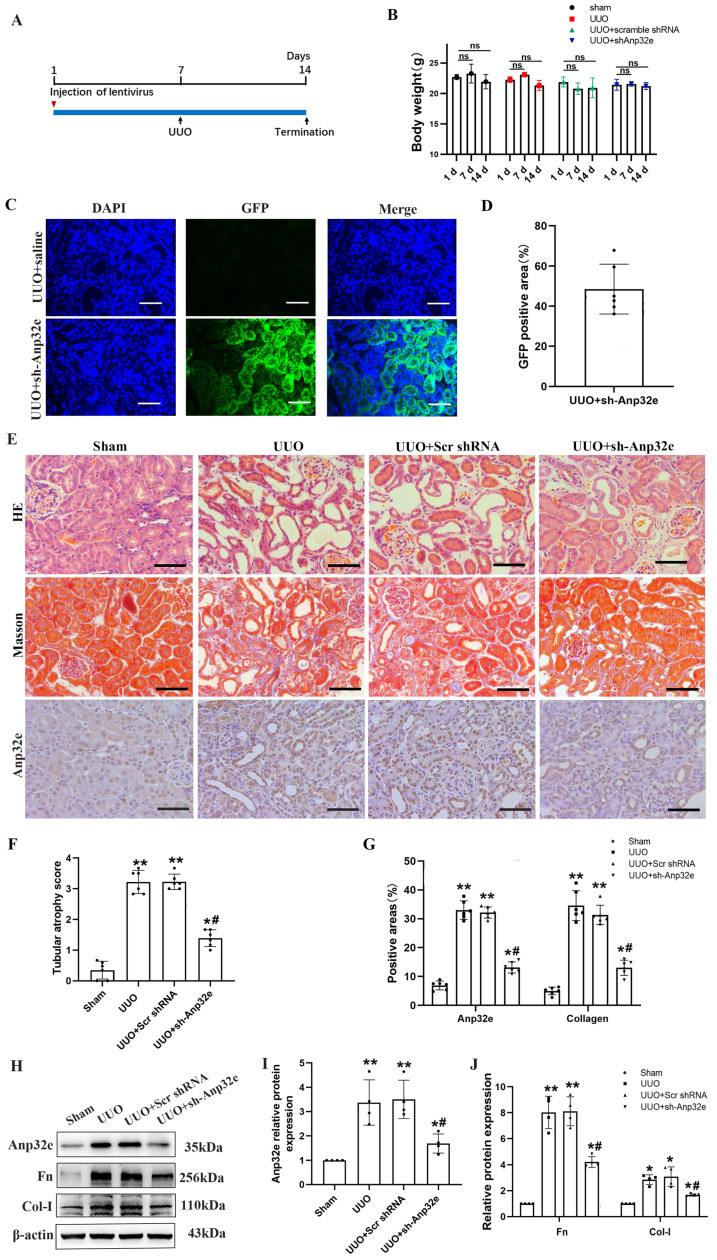
** The knockdown of Anp32e alleviated RIF in UUO mice. (A)** Mice were treated with an intrarenal injection of either sh-Anp32e or Scr shRNA 7 days before UUO surgery. Animals were euthanized 7 days after UUO. **(B)** Body weight change of each group injection (n=6). **(C, D)** Immunofluorescence of GFP protein and the area of GFP fluorescence, blue staining shows the nucleus (×400). Scale bar, 50μm, (n=6). **(E)** HE, Masson's trichrome staining and IHC analysis of Anp32e protein in various groups (×400). Scale bar, 50 μm.** (F)** Tubular atrophy score (n=6). **(G)** The positive areas (%) of Anp32e and collagen in various groups (n=6).** (H-J)** Western blotting and densitometry analysis of Anp32e, Fn and Col-I proteins in various groups (n=4).^ *^P < 0.05,^ **^P < 0.01* vs.* Sham group, ^#^P < 0.05 *vs.* UUO + Scr shRNA group. Data represent means ± SD.

To further confirm that Anp32e promotes fibrosis, we knocked down Anp32e in both *in vivo* and *in vitro* RIF models. *In vivo*, we used a lentivirus to knockdown Anp32e in the kidneys of experimental mice by intrarenal injection. UUO surgery was then performed 7 days after injection. Mice in the control groups received UUO surgery following the injection of empty lentivirus (Scr shRNA) or saline. We monitored body weight changes after lentivirus injection and found that the injection of Anp32e shRNA lentivirus did not affect body weight (Figure 5A, B).

Immunofluorescence analysis showed that approximately 50% of the renal tubules expressed GFP after lentivirus injection; there were no GFP signals in the kidneys of UUO mice injected with saline (Figure 5C, D). HE and Masson's trichrome staining and IHC staining of Anp32e showed that Anp32e protein knockdown alleviated UUO-induced renal injury and collagen deposition (Figure 5E-G). Western blotting showed that the knockdown of Anp32e protein partially suppressed the deposition of Fn and Col-I induced by UUO (Figure 5H-J).

**Figure 6:**
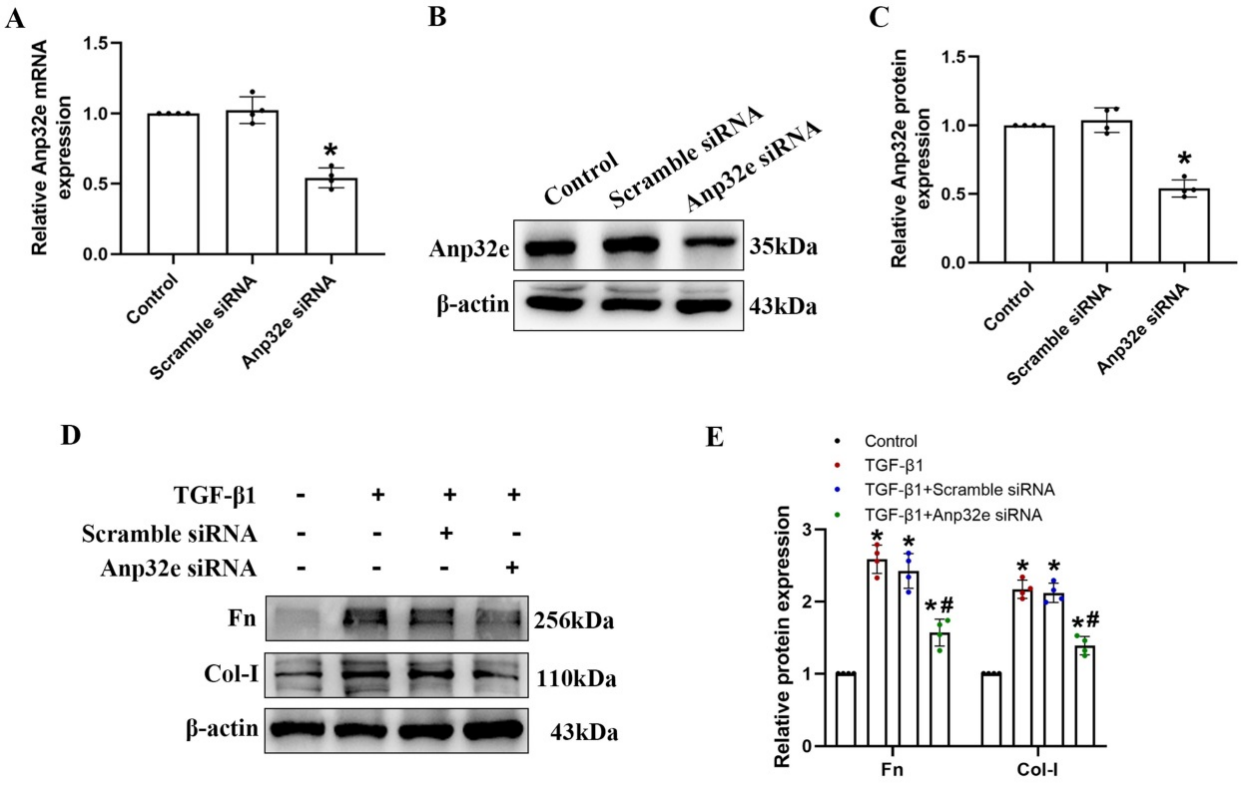
** The knockdown of Anp32e reduced the expression of fibrosis-related proteins in TGF-β1 stimulated BUMPT cells. (A-C)** qRT-PCR, western blotting and densitometry analysis of Anp32e in BUMPT cells transfected with scramble siRNA or Anp32e siRNA (n=4). **(D, E)** Western blotting and densitometry analysis of Fn and Col-I proteins in BUMPT cells transfected with scramble siRNA or Anp32e siRNA and then treated with or without TGF-β1 (n=4). ^*^P < 0.05 *vs.* scramble siRNA group, ^#^P < 0.05 *vs.* Scramble siRNA+ TGF-β1 group. Data represent means ± SD.

*In vitro*, we used Anp32e siRNA to silence the expression of Anp32e in BUMPT cells. qRT-PCR and western blotting showed that Anp32e was efficiently downregulated in BUMPT cells (Figure 6A-C). Subsequently, we used 5 ng/ml of TGF-β1 to stimulate BUMPT cells 24 h after transfection. Western blotting showed that the knockdown of Anp32e reduced the expression levels of Fn and Col-I in TGF-β1 stimulated BUMPT cells (Figure 6D, E).

### The overexpression of Anp32e promoted the deposition of fibrosis-related proteins in normal mice kidneys and BUMPT cells without TGF-β1 stimulation

**Figure 7:**
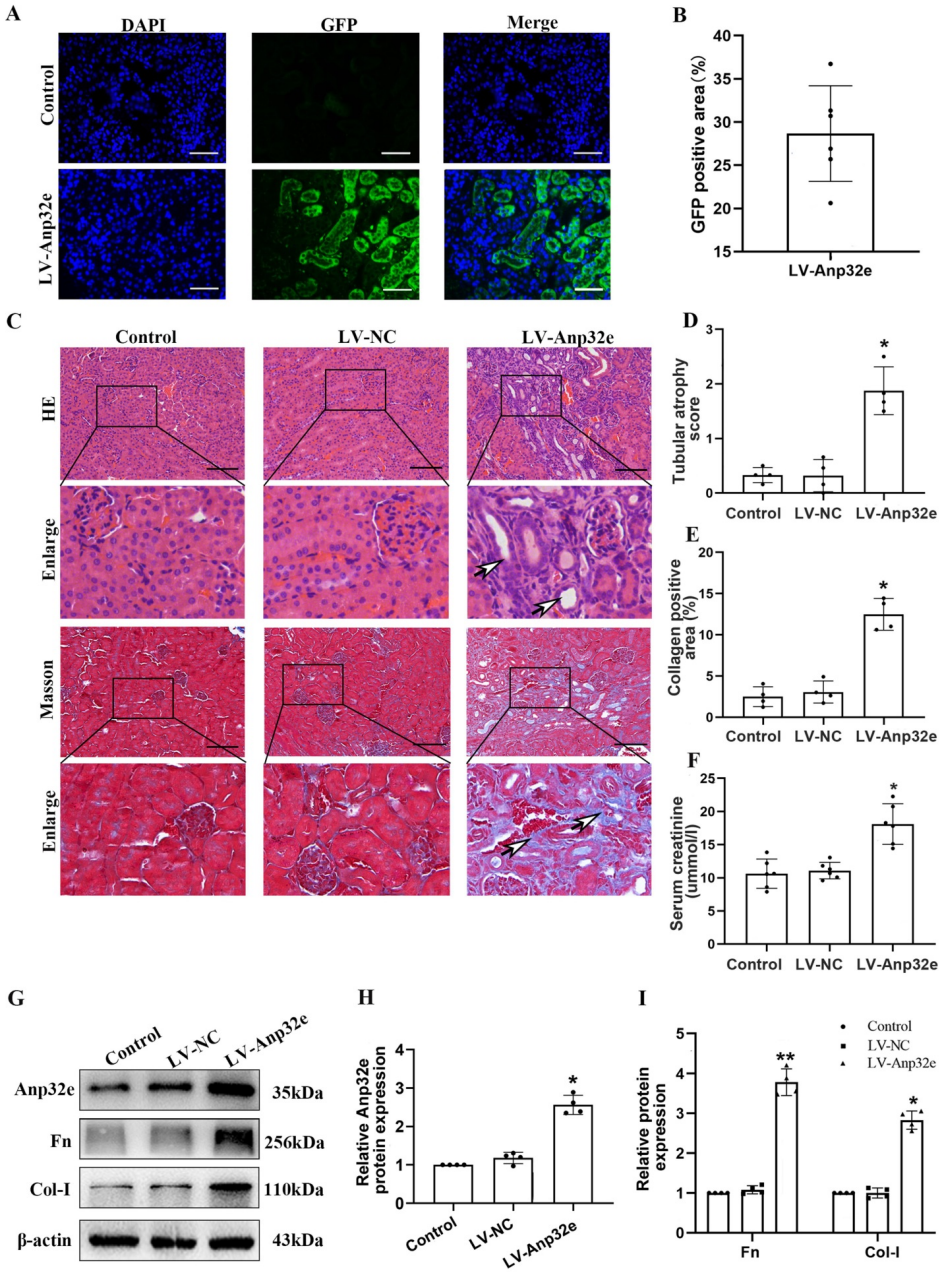
** Anp32e promoted the deposition of fibrosis-related proteins in the kidneys of experimental mice. (A, B)** Immunofluorescence of GFP protein and the area of GFP fluorescence; blue staining shows the nucleus (×400). Scale bar, 50 μm, (n=6). **(C)** HE and Masson's trichrome staining in various groups (×200). Scale bar, 100 μm, (n=4), the white arrows represent pathological changes. **(D)** Tubular atrophy score (n=4).** (E)** The positive areas (%) of collagen in various groups (n=4). **(F)** Serum creatinine in each group (n=6). **(G-I)** Western blotting and densitometry analysis of Anp32e, Fn, and Col-I proteins in various groups (n=4). ^*^P < 0.05, ^**^P <0.01 *vs.* LV-NC. Data represent means ± SD.

To confirm whether Anp32e functions as a pro-fibrotic factor, we overexpressed Anp32e alone in the kidneys of normal mice and BUMPT cells without TGF-β1 stimulation. We used a lentivirus to overexpress Anp32e protein in the kidneys of mice. Immunofluorescence analysis showed that approximately 30% of renal tubules expressed GFP after the injection of lentivirus; there were no GFP signals in the kidneys of mice in the control group (Figure 7A, B); these data indicated that LV-Anp32e had been successfully injected into the renal cortex. HE and Masson's trichrome staining indicated that compared with the empty vector lentivirus (LV-NC) group, there was obvious renal tubular cell injury and interstitial collagen deposition in the renal cortex of the LV-Anp32e group (Figure 7C-E). Furthermore, the concentration of serum creatinine in the LV-Anp32e group was significantly higher than in the LV-NC group and control group (Figure 7F). Western blotting showed that the overexpression of Anp32e alone promoted the deposition of Fn and Col-I (Figure 7G-I).

**Figure 8:**
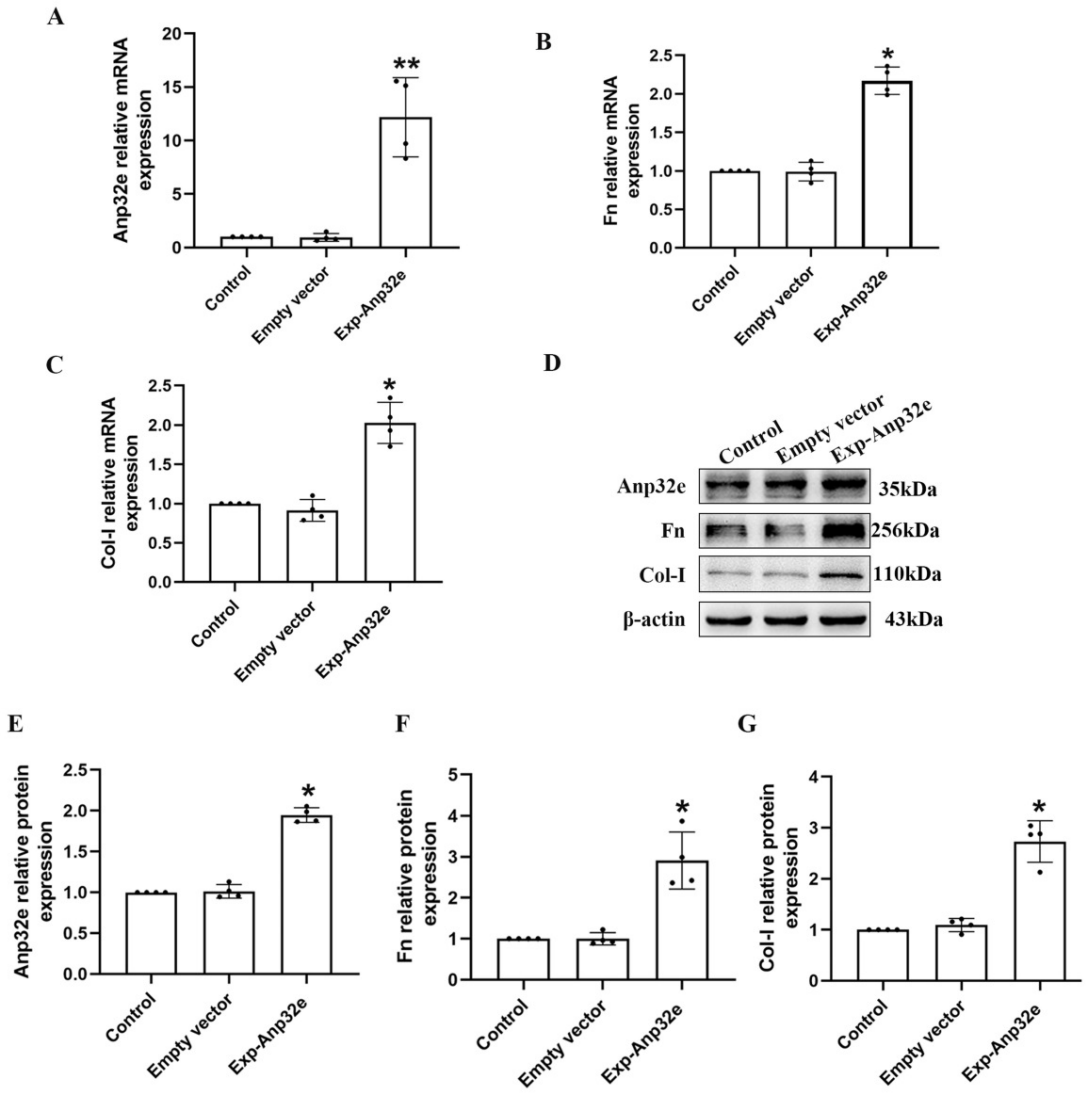
** Anp32e induced the expression of fibrosis-related proteins in BUMPT cells. (A-C)** qRT-PCR analysis of Anp32e, Fn and Col-I mRNA in BUMPT cells transfected with empty vector or Exp-Anp32e plasmids (n=4). **(D-G)** Western blotting and densitometry analysis of Anp32e, Fn, and Col-I proteins in BUMPT cells transfected with empty vector or Exp-Anp32e plasmids (n=4). ^*^P < 0.05, ^**^P <0.01 *vs*. empty vector group. Data represent means ± SD.

In addition, we overexpressed Anp32e in BUMPT cells without TGF-β1 stimulation. qRT-PCR and western blotting showed that compared with the empty vector plasmid group, the mRNA and protein levels of Fn and Col-I were both increased in Exp-Anp32e plasmid group (Figure 8). These data suggested that the overexpression of Anp32e alone could induce the deposition of fibrosis-related proteins in BUMPT cells without TGF-β1 stimulation.

### Anp32e knockdown reduced the expression of TGF-β1 and p-Smad3 proteins in UUO mice

**Figure 9:**
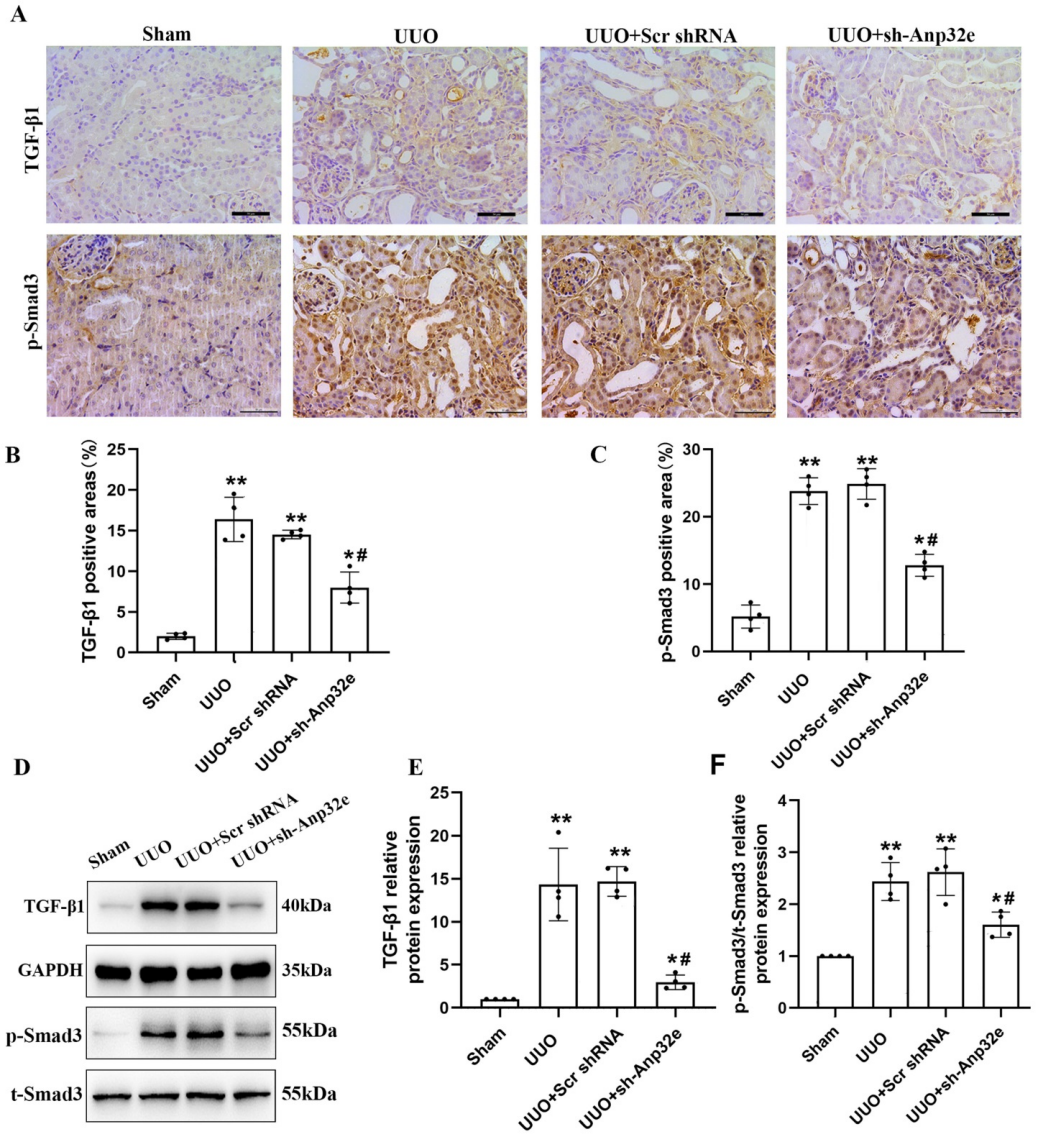
** The knockdown of Anp32e inhibited TGF-β1 expression and Smad3 phosphorylation in UUO mice. (A-C)** IHC analysis and the positive areas (%) of TGF-β1 and p-Smad3 staining in various groups (×400). Scale bar, 50 μm, (n=4). **(D-F)** Western blotting and densitometry analysis of TGF-β1 and p-Smad3 proteins in various groups (n=4).^ *^P < 0.05,^ **^P < 0.01* vs.* Sham group, ^#^P < 0.05 *vs.* UUO + Scr shRNA group. Data represent means ± SD.

To confirm the relationship between Anp32e and TGF-β1/Smad3 signaling, we investigated the effect of Anp32e knockdown on TGF-β1 expression and Smad3 phosphorylation in UUO mice kidneys. IHC staining and western blotting showed that the expression of TGF-β1 and p-Smad3 proteins were increased in the UUO group when compared with the sham group, whereas the knockdown of Anp32e inhibited UUO-induced TGF-β1 expression and Smad3 phosphorylation (Figure 9), thus indicating that Anp32e may promote renal interstitial fibrosis by regulating TGF-β1/Smad3 signaling.

### The overexpression of Anp32e promoted the deposition of fibrosis-related proteins by activating the TGF-β1/Smad3 pathway in BUMPT cells

**Figure 10:**
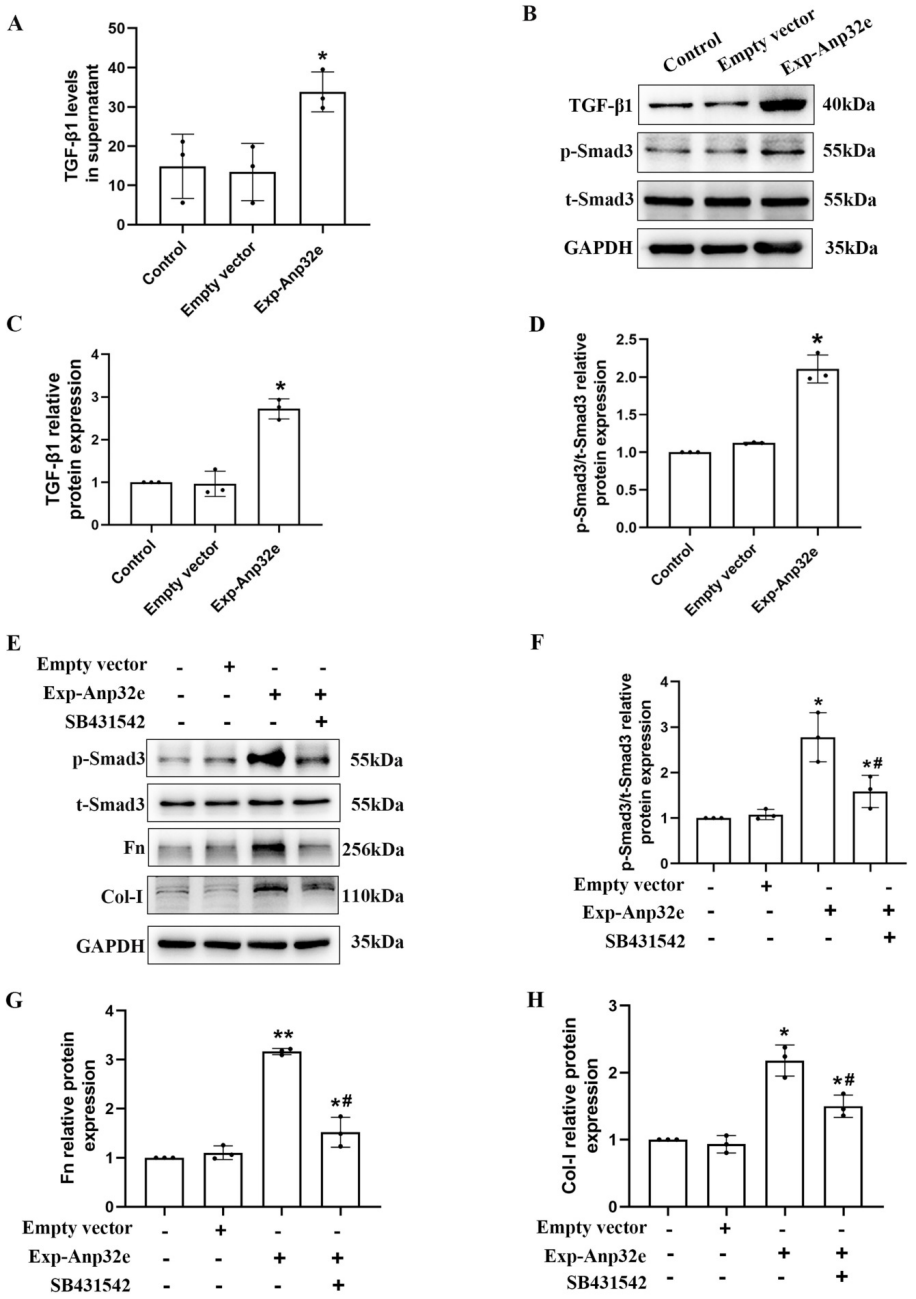
** Anp32e activated the TGF-β1/Smad3 pathway in BUMPT cells. (A)** ELISA analysis of TGF-β1 levels in BUMPT cells transfected with empty vector or Exp-Anp32e plasmids (n=3). **(B-D)** Western blotting and densitometry analysis of TGF-β1, t-Smad3 and p-Smad3 proteins in BUMPT cells transfected with empty vector or Exp-Anp32e plasmids (n=3). **(E-H)** Western blotting and densitometry analysis of t-Smad3, p-Smad3, Fn, and Col-I proteins in BUMPT cells transfected with empty vector or Exp-Anp32e plasmids, treated with or without SB431542 (n=3). ^*^P < 0.05, ^**^P <0.01 *vs*. empty vector group, ^#^P < 0.05 *vs*. Exp-Anp32e group. Data represent means ± SD.

To investigate the underlying mechanism of Anp32e regulates RIF, we transfected BUMPT cells with an Anp32e overexpressed plasmid; cells transfected with the empty plasmid as control. ELISA assays showed that the TGF-β1 levels in culture supernatant were increased in Anp32e overexpressed BUMPT cells (Figure 10A). Western blotting showed that the overexpression of Anp32e increased the expression levels of TGF-β1and p-Smad3 in BUMPT cells (Figure 10B-D). Notably, the upregulation of p-Smad3, Fn and Col-I in BUMPT cells overexpressing Anp32e was significantly attenuated by SB431542 treatment (Figure 10E-H). These results suggest that Anp32e promotes fibrosis-related protein expression by activating the TGF-β1/Smad3 signaling pathway.

## Discussion

Chronic kidney disease (CKD) is a serious global public health problem and is associated with numerous late complications that can damage many systems of the human body. Consequently, CKD creates a heavy health and economic burden 21, 22. As a common pathological change associated with various CKDs, RIF is characterized by the activation and accumulation of fibroblasts and myofibroblasts along with the extensive deposition of fibrosis-related proteins in the renal interstitium, including Fn and Col-I 2, 3, 23. Consequently, there is an urgent need to identify effective targets to suppress RIF.

Anp32e is a specific histone H2A.Z chaperone that regulates the transcription of target genes by mediating the dissociation of H2A.Z from nucleosomes 24, 25. Furthermore, Anp32e is also involved in the repair of double-strand breaks in DNA 8, 26. Several studies have shown that Anp32e participates in the occurrence and development of tumors. For example, in triple-negative breast cancer, Anp32e is known to enhance the expression of transcription factor E2F1 and induce tumorigenesis 27. In thyroid cancer, the expression of Anp32e is upregulated and promotes the proliferation and migration of thyroid cancer cells by activating the AKT/mTOR/HK2 signaling pathway 14. Similarly, Anp32e is also involved in the occurrence of pancreatic cancer and lung cancer 13, 28. Another study showed that in breast cancer, the downregulation of Anp32e inhibits the proliferation, migration, and invasion of tumor cells by blocking EMT 15, indicating that Anp32e promotes the occurrence of EMT, which is an important mechanism underlying RIF 29, 30. In the present study, we aimed to confirm that Anp32e is a pro-fibrotic factor.

Our results showed that Anp32e protein was upregulated in the renal tubular cells of IgAN patients with RIF, UUO mice and in cultured mouse proximal tubular cells exposed to TGF-β1. Moreover, we found that the overexpression of Anp32e enhanced TGF-β1-induced production of fibrosis-related proteins in BUMPT cells whereas the knockdown of Anp32e suppressed the deposition of these fibrosis-related proteins in UUO mice and TGF-β1 stimulated BUMPT cells. In particular, we found that the overexpression of Anp32e alone induced the accumulation of fibrosis-related proteins in the kidneys of mice without UUO surgery and renal proximal tubular cells without TGF-β1 stimulation. Furthermore, we confirmed that Anp32e promotes the deposition of fibrosis-related proteins by activating the TGF-β1/Smad3 signaling pathway *in vitro*.

Our results suggest that Anp32e is an important pro-fibrotic factor that contributes to RIF by activating the TGF-β1/Smad3 signaling pathway. However, the molecular mechanisms by which Anp32e activates the TGF-β/Smad3 signaling pathway have yet to be elucidated. According to previous reports, Anp32e is a specific molecular chaperone of histone variant H2A.Z, that regulates gene transcription by affecting the localization of H2A.Z in nucleosomes^24^-^26^. In Madin-Daby canine kidney cells, silencing H2A.Z promoted the expression of TGF-β1 31. These findings suggest that Anp32e may activate the TGF-β1 pathway by regulating the localization of H2A.Z in the nucleus. However, this possibility requires further research.

## Conclusion

**Figure 11:**
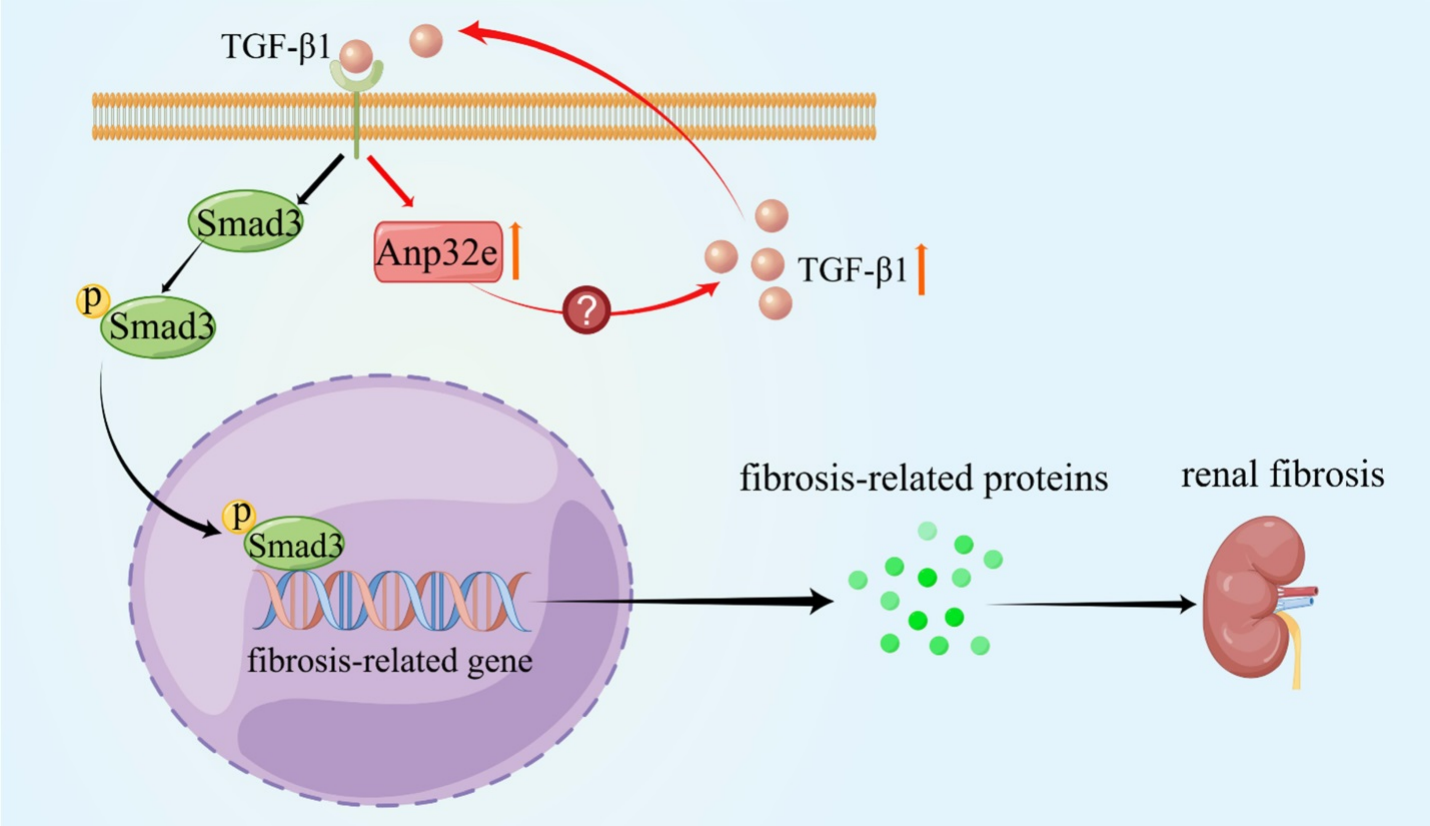
** Anp32e promotes the deposition of fibrosis-related proteins by regulating TGF-β1/Smad3 signaling** (By Figdraw). The expression of Anp32e was increased in renal tubular epithelial cells when stimulated by TGF-β1, and the upregulation of Anp32e also promoted TGF-β1 expression. TGF-β1 bound to its receptor and promoted phosphorylation of the downstream protein Smad3, which translocated into the nucleus and activated the fibrosis-related genes promoter, resulted in increased the expression of fibrosis-related proteins and leading to renal interstitial fibrosis.

As summarized in Figure 11, we demonstrate that Anp32e acts as a major regulator of RIF by promoting the deposition of fibrosis-related proteins in the renal interstitium by activating the TGF-β1/Smad3 signaling pathway.

## Figures and Tables

**Table 1:** Primer sequences for qRT‐PCR.

Gene	Forward	Reverse
Anp32e	GGTAGTGGGCGTTCGTCTTT	CAGGCCTTCGATTTCCCCAT
Fn	GTGGCTGCCTTCAACTTCTC	TTGCAAACCTTCAATGGTCA
Col-I	ATGTCGCTATCCAGCTGACC	CCTTCTTGAGGTTGCCAGTC
β-actin	TCACCCACACTGTGCCCATCATCGA	CAGCGGAACCGCTCATTGCCAATGG

**Table 2:** Clinical Characteristics of the IgAN Group and Control Group (case %, X±SD).

	IgAN group (n=9)	Control group (n=7)	P values
Male Sex (number)	6 (66.7%)	3 (42.8%)	0.615
Average age (years)	42±7.57	32.78±9.95	0.062
Blood urea nitrogen (mmol/L)	7.50±2.82	4.54±1.41	0.024*
Serum creatinine (umol/L)	126.50±50.89	58.53±11.02	0.004**
Blood uric acid (umol/L)	358.51±151.02	302.41±67.73	0.379
Proteinuria (case)	9 (100%)	0 (0%)	0.000087**

*P < 0.05, **P < 0.01

## Abbreviations

Anp32e: Acidic nuclear phosphoprotein 32 family member e
RIF: renal interstitial fibrosis
CKD: chronic kidney disease
EMT: epithelial-mesenchymal transition
UUO: unilateral ureteral obstruction
BUMPT: the Boston University mouse proximal tubular cell line
TGF: transforming growth factor
IgAN: IgA nephropathy
Fn: fibronectin
Col-I: collagen type I
sh-Anp32e: Anp32e shRNA lentivirus
Scr-shRNA: scramble shRNA lentivirus
LV-Anp32e: Anp32e overexpressing lentivirus
LV-NC: empty vector lentivirus
Scr siRNA: scramble siRNA
DMEM: Dulbecco's Modified Eagle's Medium
HE: hematoxylin-eosin
IHC: Immunohistochemistry
qRT-PCR: quantitative real-time PCR
